# Supplementary material for: Association of socioeconomic deprivation with asthma care, outcomes, and deaths in Wales: A 5-year national linked primary and secondary care cohort study
Source: PLoS Med. 2021 Feb 12;18(2):e1003497. doi: 10.1371/journal.pmed.1003497 (PMC7880491; doi:10.1371/journal.pmed.1003497)
Supplement: S1 Text — (PDF) [file pmed.1003497.s002.pdf]

S1 Text: Study design and data sources

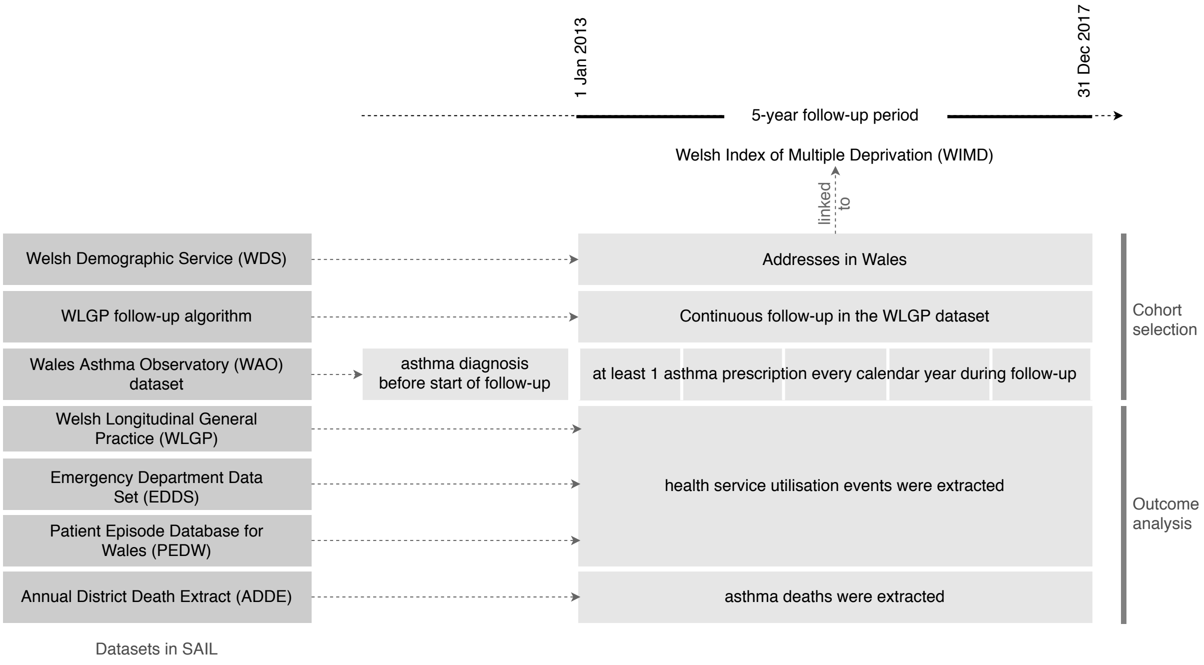

.

Datasets used in this study included:

- **The Welsh Demographic Service (WDS)** contains de-identified demographic and administrative information for National Health Services (NHS) patient in Wales.
- **The Welsh Longitudinal General Practice (WLGP) dataset** (the "2018-08-20" extract) contains primary care records coded in Read codes from about 76% of general practices in Wales.
- **The Emergency Department Data Set (EDDS) for Wales** captures attendances to emergency department (ED) and minor injury unit in NHS hospitals in Wales since 2009 [1]. Collected data include diagnostic investigations, diagnosis, affected anatomical areas, treatment provided, and other administrative information about the attendance. Primary diagnosis and up to five secondary diagnoses can be recorded, with 83 broad diagnostic categories available.
- **The Patient Episode Database for Wales (PEDW)** captures all inpatient admissions to NHS Wales hospitals as well as most admissions of Welsh residents to hospitals in England [2]. Collected data include admission diagnoses (one primary diagnosis and up to 13 secondary diagnoses recorded in ICD-10), procedures and operations performed during admissions, length of stay (LOS), and Healthcare Resource Groups (HRGs) cost codes.
- **The Annual District Death Extract (ADDE) dataset** is produced and maintained by the Office for National Statistics (ONS) and is linked to the SAIL Databank. It contains mortality data since 1996 including up to eight causes of death from Medical Certificates of Cause of Death (MCCD) certified by a medical practitioner or a coroner.[*] Causes of death are automatically or manually coded using 10th

[*]Mortality Statistics: Metadata, July 2015, Office for National Statistics (link, accessed 9 Dec 2019).

revision of the International Classification of Disease (ICD-10) from the MCCDs.

- **The Wales Asthma Observatory (WAO)** is a data resource derived from the above datasets and includes patients identified using a variety of asthma case definitions as well as asthma-related research variables [3].

MA has full access to the aforementioned data sources.

References

1 Lyons RA, Turner S, Lyons J, Walters A, Snooks HA, Greenacre J, et al. All Wales Injury Surveillance System revised: development of a population-based system to evaluate single-level and multilevel interventions. *Inj Prev*. 2016;**22**(Suppl 1): i50–i55.

2 NHS Wales Data Dictionary - Admitted Patient Care Data Set (APC Ds) - Data Set Structure. URL: `http://www.datadictionary.wales.nhs.uk/#!WordDocuments/datasetstructure.htm` (visited on Mar. 21, 2020).

3 Al Sallakh MA. Creating and utilising the Wales Asthma Observatory to support health policy, health service planning and clinical research. PhD thesis. Swansea University, 2018.
