# Supplementary material for: Association of socioeconomic deprivation with asthma care, outcomes, and deaths in Wales: A 5-year national linked primary and secondary care cohort study
Source: PLoS Med. 2021 Feb 12;18(2):e1003497. doi: 10.1371/journal.pmed.1003497 (PMC7880491; doi:10.1371/journal.pmed.1003497)
Supplement: S2 Text — (PDF) [file pmed.1003497.s003.pdf]

S2 Text: Patient selection

The source population included people who:

- Had records in the Welsh Demographic Service (WDS) and the WLGP datasets;
- Were born before 1 January, 2013; and
- Lived in Welsh addresses between 1 January, 2013 and 31 December, 2017.

The study cohort included people in the source population who:

- Lived at least until 31 December, 2017;
- Had continuous follow-up in the primary care dataset over the follow-up period; [1]
- Had a GP-recorded asthma diagnosis before 1 January, 2013;
- Received at least one asthma prescription in each of the five follow-up years.

We ascertained asthma diagnosis and prescriptions by linking to the Wales Asthma Observatory, [2] a tagged asthma cohort in SAIL.

Duplicate records in each dataset have been removed, and addresses with missing LSOA codes have been excluded.

Patient section is visualised in the following flowchart:

Flowchart of patient selection and frequencies

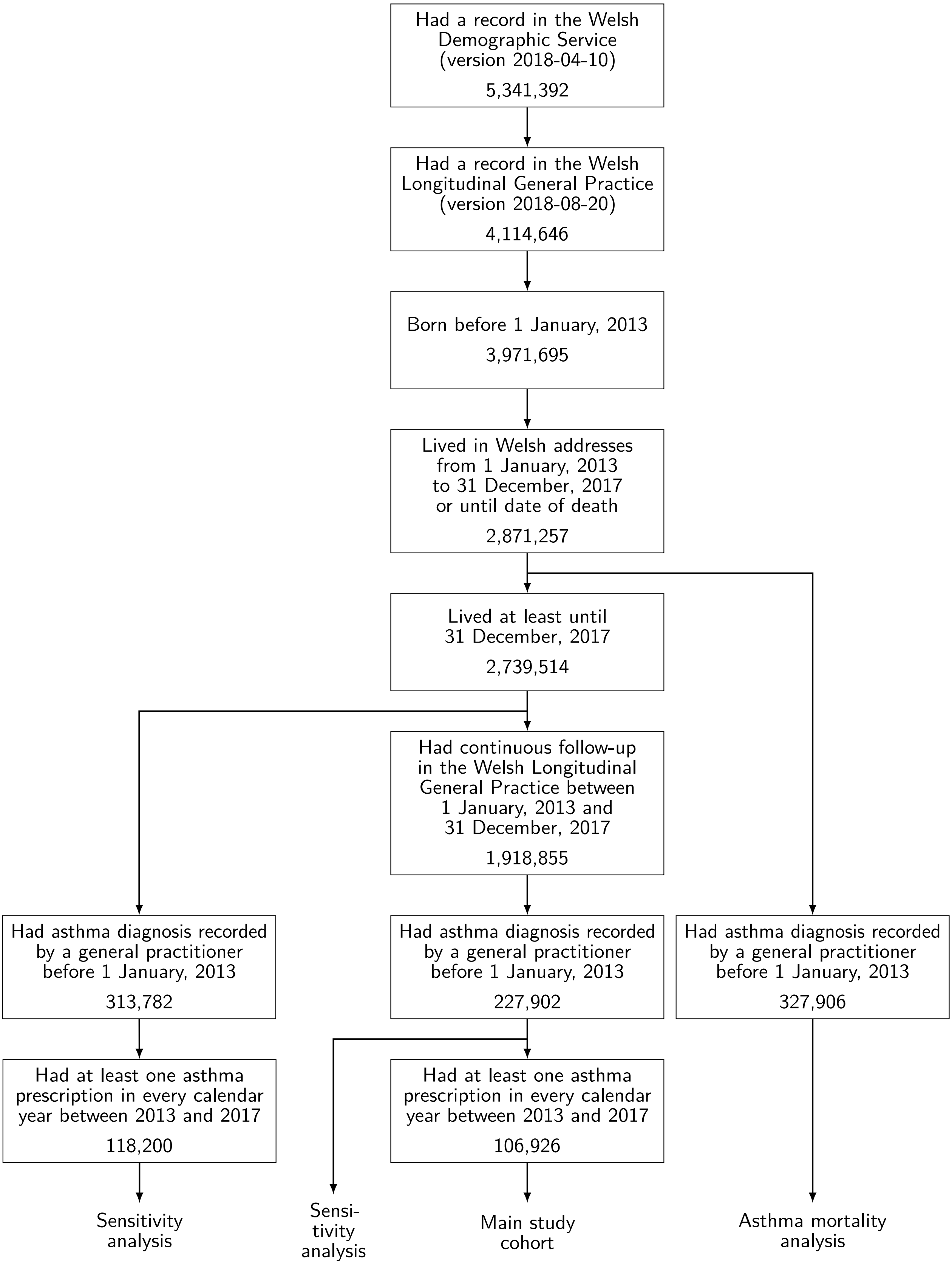

References

1 Thayer D, Rees A, Kennedy J, Collins H, Harris D, Halcox J, et al. Measuring follow-up time in routinely-collected health datasets: Challenges and solutions. *PLoS One*. 2020;**15**(2): 1–11.

2 Al Sallakh MA. Creating and utilising the Wales Asthma Observatory to support health policy, health service planning and clinical research. PhD thesis. Swansea University, 2018.
