# Supplementary material for: Association of socioeconomic deprivation with asthma care, outcomes, and deaths in Wales: A 5-year national linked primary and secondary care cohort study
Source: PLoS Med. 2021 Feb 12;18(2):e1003497. doi: 10.1371/journal.pmed.1003497 (PMC7880491; doi:10.1371/journal.pmed.1003497)

S3 Text: Sensitivity analysis

Relaxing the section criteria to include patients with no continuous asthma prescriptions:

The main findings of the study did not significantly change by repeating the analysis on a wider cohort of patients with ever-diagnosed asthma, regardless of having continuous asthma treatment every year (see the forest plots below). In this more inclusive cohort (n = 227,902), the observed socioeconomic gap in emergency care between the most and least deprived quintiles widened slightly: the IRRs increased for asthma-related A&E attendances from 1.269 (1.103 to 1.459) to 1.371 (1.210 to 1.553, p-value < 0.001), for total asthma hospitalisations from 1.977 (1.740 to 2.246) to 2.244 (1.996 to 2.523, p-value < 0.001), for asthma emergency hospitalisations from 1.559 (1.385 to 1.756) to 1.793 (1.607 to 2.000, p-value < 0.001), and for length of stay from 1.640 (1.385 to 1.942) to 2.004 (1.720 to 2.335, p-value < 0.001), whereas for total asthma-related primary care consultations the ratio inverted from 0.981 (0.972 to 0.991) to 1.055 (1.039 to 1.071, p-value < 0.001), and for asthma reviews the ratio was inverted from 0.980 (0.970 to 0.990) to 1.064 (1.048 to 1.079, p-value < 0.001).

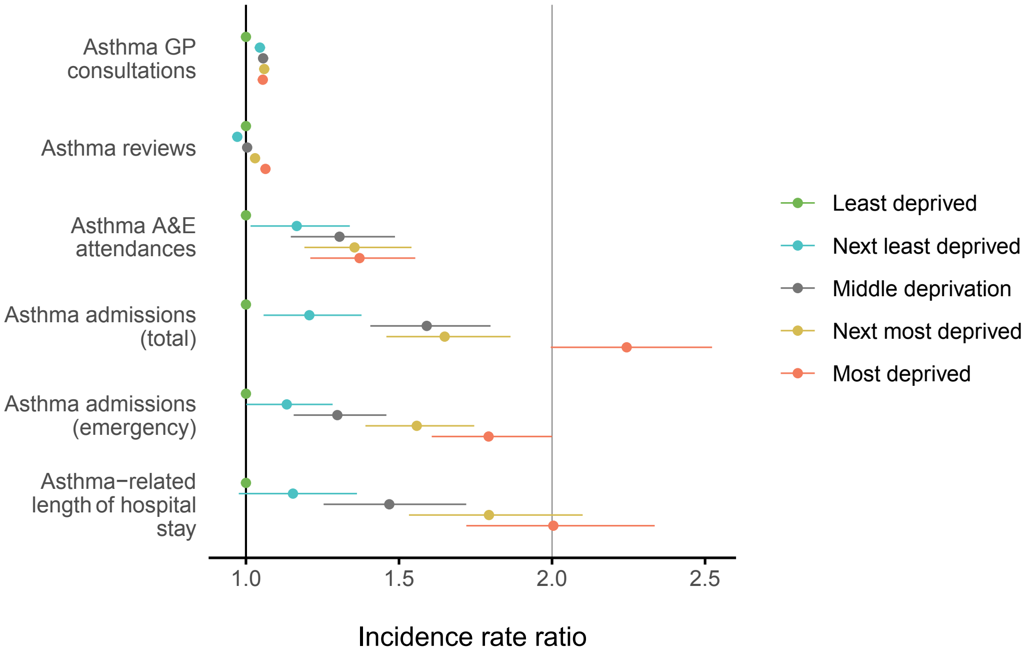

Relaxing the section criteria to include patients with no continuous followup in the primary care dataset:

By dropping the condition of continuous follow-up in the primary care dataset over the study period from the patient selection criteria, the study cohort would have included 118,200 patients, among which 11,274 (9.5%) did not have continuous follow-up. However, in this less restrictive cohort, the IRR patterns across the WIMD quintiles showed minor changes; between the deprivation extremes, the IRRs for asthma-related GP consultations became 0.983 (0.974 to 0.992, p-value $<$ 0.001), for asthma reviews 0.973 (0.963 to 0.983, p-value $<$ 0.001), for ED attendances 1.355 (1.187 to 1.546, p-value $<$ 0.001), for all asthma hospitalisations 1.998 (1.772 to 2.254, p-value $<$ 0.001), for emergency asthma hospitalisations 1.624 (1.452 to 1.817, p-value $<$ 0.001), and for length of stay 1.716 (1.464 to 2.012, p-value $<$ 0.001).

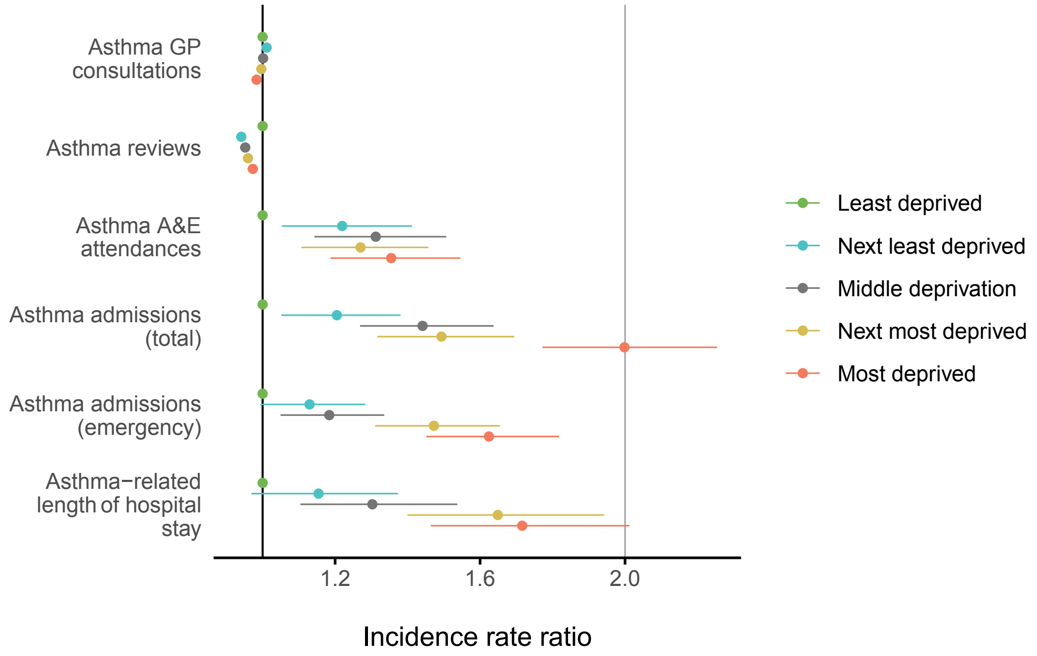

Supplement: S3 Text — (PDF) [file pmed.1003497.s004.pdf]
