# Supplementary material for: Association of socioeconomic deprivation with asthma care, outcomes, and deaths in Wales: A 5-year national linked primary and secondary care cohort study
Source: PLoS Med. 2021 Feb 12;18(2):e1003497. doi: 10.1371/journal.pmed.1003497 (PMC7880491; doi:10.1371/journal.pmed.1003497)
Supplement: S1 Table — (PDF) [file pmed.1003497.s005.pdf]

# S1 Table: Clinical code sets

| Code | Description |
|---|---|
| Asthma diagnosis (Read codes) | |
| H33.. | Asthma |
| H330. | Extrinsic (atopic) asthma |
| H3300 | Extrinsic asthma without status asthmaticus |
| H3301 | Extrinsic asthma with status asthmaticus |
| H330z | Extrinsic asthma NOS |
| H331. | Intrinsic asthma |
| H3310 | Intrinsic asthma without status asthmaticus |
| H3311 | Intrinsic asthma with status asthmaticus |
| H331z | Intrinsic asthma NOS |
| H332. | Mixed asthma |
| H333. | Acute exacerbation of asthma |
| H334. | Brittle asthma |
| H335. | Chronic asthma with fixed airflow obstruction |
| H33z. | Asthma unspecified |
| H33z0 | Status asthmaticus NOS |
| H33z1 | Asthma attack |
| H33z2 | Late-onset asthma |
| H33zz | Asthma NOS |
| H3120 | Chronic asthmatic bronchitis |
| 1O2.. | Asthma confirmed |
| Asthma GP consultations (Read codes) | |
| 173c. | Occupational asthma |
| 173d. | Work aggravated asthma |
| 178.. | Asthma trigger |
| 1780. | Aspirin induced asthma |
| 1781. | Asthma trigger - pollen |
| 1782. | Asthma trigger - tobacco smoke |
| 1783. | Asthma trigger - warm air |
| 1784. | Asthma trigger - emotion |
| 1785. | Asthma trigger - damp |
| 1786. | Asthma trigger - animals |
| 1787. | Asthma trigger - seasonal |
| 1788. | Asthma trigger - cold air |
| 1789. | Asthma trigger - respiratory infection |
| 178A. | Asthma trigger - airborne dust |
| 178B. | Asthma trigger - exercise |
| 1O2.. | Asthma confirmed |
| 388t. | Royal College of Physicians asthma assessment |
| 38DL. | Asthma control test |
| 38DV. | Mini asthma quality of life questionnaire |
| 38QM. | Childhood Asthma Control Test |
| 661M1 | Asthma self-management plan agreed |
| 661N1 | Asthma self-management plan review |
| 663d. | Emergency asthma admission since last appointment |
| 663e. | Asthma restricts exercise |
| 663e0 | Asthma sometimes restricts exercise |
| 663e1 | Asthma severely restricts exercise |
| 663f. | Asthma never restricts exercise |
| 663h. | Asthma - currently dormant |
| 663j. | Asthma - currently active |
| 663m. | Asthma accident and emergency attendance since last visit |
| 663n. | Asthma treatment compliance satisfactory |
| 663N. | Asthma disturbing sleep |
| 663N0 | Asthma causing night waking |
| 663N1 | Asthma disturbs sleep weekly |
| 663N2 | Asthma disturbs sleep frequently |
| 663O. | Asthma not disturbing sleep |
| 663O0 | Asthma never disturbs sleep |
| 663p. | Asthma treatment compliance unsatisfactory |
| 663P. | Asthma limiting activities |
| 663P0 | Asthma limits activities 1 to 2 times per month |
| 663P1 | Asthma limits activities 1 to 2 times per week |
| 663P2 | Asthma limits activities most days |
| 663q. | Asthma daytime symptoms |
| 663Q. | Asthma not limiting activities |
| 663r. | Asthma causes night symptoms 1 to 2 times per month |
| 663s. | Asthma never causes daytime symptoms |
| 663t. | Asthma causes daytime symptoms 1 to 2 times per month |
| 663u. | Asthma causes daytime symptoms 1 to 2 times per week |
| 663U. | Asthma management plan given |
| 663v. | Asthma causes daytime symptoms most days |
| 663V. | Asthma severity |
| 663V0 | Occasional asthma |
| 663V1 | Mild asthma |
| 663V2 | Moderate asthma |
| 663V3 | Severe asthma |
| 663w. | Asthma limits walking up hills or stairs |
| 663W. | Asthma prophylactic medication used |
| 663x. | Asthma limits walking on the flat |
| 663y. | Number of asthma exacerbations in past year |
| 66Y5. | Change in asthma management plan |
| 66Y9. | Step up change in asthma management plan |
| 66YA. | Step down change in asthma management plan |
| 66YC. | Absent from work or school due to asthma |
| 66YE. | Asthma monitoring due |
| 66YJ. | Asthma annual review |
| 66YK. | Asthma follow-up |
| 66Yp. | Asthma review using Royal College of Physicians three questions |
| 66YP. | Asthma night-time symptoms |
| 66YQ. | Asthma monitoring by nurse |
| 66Yq.. | Asthma causes night time symptoms 1 to 2 times per week |
| 66Yr. | Asthma causes symptoms most nights |
| 66YR. | Asthma monitoring by doctor |
| 66Ys. | Asthma never causes night symptoms |
| 66Yu. | Number of days absent from school due to asthma in past 6 months |
| 66YZ. | Does not have asthma management plan |
| 679J. | Health education - asthma |
| 679J0 | Health education - asthma self management |
| 679J1 | Health education - structured asthma discussion |
| 679J2 | Health education - structured patient focused asthma discussion |
| 8791. | Further asthma - drug prevent. |
| 8793. | Asthma control step 0 |
| 8794. | Asthma control step 1 |
| 8795. | Asthma control step 2 |
| 8796. | Asthma control step 3 |
| 8797. | Asthma control step 4 |
| 8798. | Asthma control step 5 |
| 8B3j. | Asthma medication review |
| 8CMA0 | Patient has a written asthma personal action plan |
| 8CR0. | Asthma clinical management plan |
| 8H2P. | Emergency admission, asthma |
| 8HTT. | Referral to asthma clinic |
| 9hA.. | Exception reporting: asthma quality indicators |
| 9hA1. | Excepted from asthma quality indicators: Patient unsuitable |
| 9N1d. | Seen in asthma clinic |
| 9N1d0 | Seen in school asthma clinic |
| 9NI8. | Asthma outreach clinic |
| 9NNX. | Under care of asthma specialist nurse |
| 9OJ.. | Asthma monitoring admin. |
| 9OJ1. | Attends asthma monitoring |
| 9OJ2. | Refuses asthma monitoring |
| 9OJ3. | Asthma monitor offer default |
| 9OJ4. | Asthma monitor 1st letter |
| 9OJ5. | Asthma monitor 2nd letter |
| 9OJ6. | Asthma monitor 3rd letter |
| 9OJ7. | Asthma monitor verbal invite |
| 9OJ8. | Asthma monitor phone invite |
| 9OJ9. | Asthma monitoring deleted |
| 9OJA. | Asthma monitoring check done |
| 9OJB. | Asthma monitoring invitation SMS (short message service) text message |
| 9OJC. | Asthma monitoring invitation email |
| 9OJZ. | Asthma monitoring admin.NOS |
| 9Q21. | Patient in asthma study |
| SLF7. | Antiasthmatic poisoning |
| SLF7z | Antiasthmatic poisoning NOS |
| Asthma Reviews (Read codes) | |
| 66YJ. | Asthma annual review |
| 66YK. | Asthma follow-up |
| 66Yp. | Asthma review using Royal College of Physicians three questions |
| 66YQ. | Asthma monitoring by nurse |
| 8B3j. | Asthma medication review |
| 9OJA. | Asthma monitoring check done |
| Asthma Emergency Department attendances (A&E code) | |
| 14A | Asthma |
| Asthma Hospitalisations (ICD-10 codes) | |
| J45 | Asthma |
| J45.0 | Predominantly allergic asthma |
| J45.1 | Nonallergic asthma |
| J45.8 | Mixed asthma |
| J45.9 | Asthma, unspecified |
| J46 | Status asthmaticus |
| Asthma Prescriptions (Read codes) | |
| Short-acting beta agonist (SABA) | |
| c11%% | SALBUTAMOL [ORAL PREPARATIONS] |
| c12%% | SALBUTAMOL [PARENTERAL PREPARATIONS] |
| c13%% | SALBUTAMOL [INHALATION PREPRATIONS] |
| c14%% | TERBUTALINE SULPHATE [RESPIRATORY USE] |
| c15%% | FENOTEROL HYDROBROMIDE |
| c1E%% | SALBUTAMOL [INHALATION PREPRATIONS 2] |
| Inhaled corticosteroid (ICS) | |
| c615. | *BECOTIDE rotahaler device |

| Code | Description |
|---|---|
| c616. | BECOTIDE 50micrograms/mL nebuliser solution |
| c617. | BECOTIDE-100 100microgram inhaler |
| c618. | *VOLUMATIC spacer device |
| c619. | BECODISK 100micrograms diskhaler 14x8 |
| c61A. | BECLOMETASONE DIPROPIONATE 400micrograms disks+disk inhaler |
| c61B. | BECLOMETASONE DIPROPIONATE 400micrograms disk refill |
| c61C. | BECLOMETHASONE DIPROPIONATE 250micrograms inhaler+spacer device |
| c61E. | BECLOMETASONE DIPROPIONATE 250micrograms breath-actuated aerosol inhaler |
| c61F. | BECLOMETASONE DIPROPIONATE 100micrograms breath-actuated aerosol inhaler |
| c61G. | *FILAIR 50micrograms inhaler |
| c61H. | *FILAIR 100micrograms inhaler |
| c61J. | FILAIR FORTE 250micrograms inhaler |
| c61K. | BECLAZONE 50micrograms inhaler |
| c61L. | BECLAZONE 100micrograms inhaler |
| c61M. | BECLAZONE 250micrograms inhaler |
| c61N. | BECLAZONE 50 EASI-BREATHE inhaler |
| c61O. | BECLAZONE 100 EASI-BREATHE inhaler |
| c61P. | BECLAZONE 250 EASI-BREATHE inhaler |
| c61Q. | BECLOFORTE INTEGRA 250micrograms inhaler+compact spacer |
| c61R. | BECLOFORTE INTEGRA 250micrograms refill |
| c61S. | BECLOMETHASONE DIPROPIONATE 250micrograms inhaler+compact spacer |
| c61T. | BECLOMETHASONE DIPROPIONATE 250micrograms compact spacer refill |
| c61U. | BECLOMETHASONE rotahaler device |
| c61V. | BECLOMETHASONE DIPROPIONATE 50micrograms vortex metered dose inhaler |
| c61W. | *BDP 50micrograms Spacehaler |
| c61X. | BECLOMETHASONE DIPROPIONATE 100micrograms vortex metered dose inhaler |
| c61Y. | *BDP 100micrograms Spacehaler |
| c61Z. | BECLOMETHASONE DIPROPIONATE 250micrograms vortex metered dose inhaler |
| c61a. | BECODISK 200micrograms diskhaler 14x8 |
| c61b. | BECOTIDE 400micrograms rotacaps |
| c61c. | BECODISK 100micrograms disk refill 14x8 |
| c61d. | BECODISK 200micrograms disk refill 14x8 |
| c61e. | BECODISK 400micrograms diskhaler 7x8 |
| c61f. | BECODISK 400micrograms disk refill 7x8 |
| c61g. | BECLOFORTE VM 250micrograms inhaler+volumatic |
| c61h. | BECLOMETASONE DIPROPIONATE 400micrograms inhalation capsules |
| c61i. | BECOTIDE-200 200microgram inhaler |
| c61j. | *AEROBEC 50microgram Autohaler |
| c61k. | AEROBEC FORTE 250micrograms Autohaler |
| c61l. | AEROBEC 100microgram Autohaler |
| c61m. | BECLOFORTE DISKHALER 400micrograms 14x8 |
| c61n. | BECLOFORTE DISKS 400micrograms disk refill 14x8 |
| c61p. | BECLOMETASONE DIPROPIONATE 100micrograms disks+disk inhaler |
| c61q. | BECLOMETASONE DIPROPIONATE 200micrograms disks+disk inhaler |
| c61r. | BECLOMETASONE DIPROPIONATE 100micrograms disk refill |
| c61s. | BECLOMETASONE DIPROPIONATE 200micrograms disk refill |
| c61u. | BECLOMETASONE DIPROPIONATE 200micrograms inhaler |
| c61v. | BECLOMETASONE DIPROPIONATE 50micrograms inhaler |
| c61w. | BECLOMETASONE DIPROPIONATE 100micrograms inhalation capsules |
| c61x. | BECLOMETASONE DIPROPIONATE 200micrograms inhalation capsules |
| c61y. | BECLOMETHASONE DIPROPIONATE 50micrograms/mL nebuliser solution |
| c61z. | BECLOMETASONE DIPROPIONATE 100micrograms inhaler |
| c62.. | BECLOMETASONE COMPOUNDS |
| c621. | *VENTIDE inhaler |
| c622. | *VENTIDE Rotacaps |
| c623. | *VENTIDE paediatric Rotacaps |
| c624. | *VENTIDE Rotahaler device |
| c63.. | *BETAMETHASONE VALERATE |
| c631. | *BEXTASOL 100microgram inhaler |
| c63z. | BETAMETHASONE 100micrograms inhaler |
| c64.. | BUDESONIDE [RESPIRATORY USE] |
| c641. | PULMICORT 200micrograms inhaler 200dose |
| c642. | PULMICORT 200micrograms refill 100dose |
| c643. | PULMICORT 200micrograms refill 200dose |
| c644. | PULMICORT LS 50micrograms inhaler |
| c645. | PULMICORT LS 50micrograms refill |
| c646. | *NEBUHALER spacer device |
| c647. | PULMICORT 200microgram inhaler 100dose |
| c649. | PULMICORT 400microgram Turbohaler 50dose |
| c64A. | BUDESONIDE 200micrograms refill cannister |
| c64B. | BUDESONIDE 50micrograms spacer inhaler |
| c64C. | PULMICORT 200micrograms spacer inhaler |
| c64D. | PULMICORT LS 50micrograms spacer inhaler |
| c64E. | PULMICORT 200micrograms inhaler with NebuChamber |
| c64F. | BUDESONIDE 200micrograms/dose dry powder cartridge refill |
| c64G. | NOVOLIZER BUDESONIDE 200micrograms/dose dry powder cartridge refill |
| c64H. | EASYHALER BUDESONIDE 100micrograms breath-actuated dry powder inhaler |
| c64I. | EASYHALER BUDESONIDE 200micrograms breath-actuated dry powder inhaler |
| c64J. | EASYHALER BUDESONIDE 400micrograms breath-actuated dry powder inhaler |
| c64K. | PULMICORT 100micrograms CFC-free inhaler |
| c64a. | PULMICORT 500micrograms Respules 2mL unit |
| c64b. | PULMICORT 1mg Respules 2mL unit |
| c64c. | PULMICORT 100microgram Turbohaler 200dose |
| c64d. | BUDESONIDE 100micrograms breath-actuated dry powder inhaler |
| c64e. | BUDESONIDE 50micrograms refill cannister |
| c64g. | BUDESONIDE 200micrograms breath-actuated dry powder inhaler |
| c64h. | BUDESONIDE 400micrograms breath-actuated dry powder inhaler |
| c64i. | BUDESONIDE 500micrograms/2mL nebuliser solution |
| c64j. | BUDESONIDE 1mg/2mL nebuliser solution |
| c64k. | *BUDESONIDE 200 Cyclocaps |
| c64l. | *BUDESONIDE 400 Cyclocaps |
| c64m. | BUDESONIDE 200micrograms inhalation capsules |
| c64n. | BUDESONIDE 400micrograms inhalation capsules |
| c64o. | BUDESONIDE 200micrograms inhaler with spacer device |
| c64p. | NOVOLIZER BUDESONIDE 200micrograms/dose dry powder cartridge and refillable inhaler device |
| c64u. | BUDESONIDE 200micrograms/dose dry powder cartridge and refillable inhaler device |
| c64v. | BUDESONIDE 200micrograms inhaler |
| c64x. | *BUDESONIDE refill 200dose |
| c64y. | BUDESONIDE 50micrograms inhaler |
| c64z. | BUDESONIDE 200micrograms spacer inhaler |
| c65.. | FLUTICASONE PROPIONATE [RESPIRATORY USE] |
| c651. | FLIXOTIDE 50micrograms diskhaler |
| c652. | FLIXOTIDE 100micrograms diskhaler |
| c653. | FLIXOTIDE 250micrograms diskhaler |
| c654. | FLUTICASONE PROPIONATE 50micrograms disks+disk inhaler |
| c655. | FLUTICASONE PROPIONATE 100micrograms disks+disk inhaler |
| c656. | FLUTICASONE PROPIONATE 250micrograms disks+disk inhaler |
| c657. | FLIXOTIDE 50micrograms disk refill |
| c658. | FLIXOTIDE 100micrograms disk refill |
| c659. | FLIXOTIDE 250micrograms disk refill |
| c65A. | FLUTICASONE PROPIONATE 50micrograms disk refill |
| c65B. | FLUTICASONE PROPIONATE 100micrograms disk refill |
| c65C. | FLUTICASONE PROPIONATE 250micrograms disk refill |
| c65D. | FLIXOTIDE 25micrograms inhaler |
| c65E. | FLIXOTIDE 50micrograms inhaler |
| c65F. | FLIXOTIDE 125micrograms inhaler |
| c65G. | FLUTICASONE PROPIONATE 25micrograms inhaler |
| c65H. | FLUTICASONE PROPIONATE 50micrograms inhaler |
| c65I. | FLUTICASONE PROPIONATE 125micrograms inhaler |
| c65K. | FLIXOTIDE 250micrograms inhaler |
| c65L. | FLIXOTIDE 500micrograms diskhaler |
| c65M. | FLIXOTIDE 500micrograms disk refill |
| c65N. | FLUTICASONE PROPIONATE 500micrograms disks+disk inhaler |
| c65O. | FLUTICASONE PROPIONATE 500micrograms disk refill |
| c65P. | FLUTICASONE PROPIONATE 50micrograms breath-actuated dry powder inhaler |
| c65Q. | FLUTICASONE PROPIONATE 100micrograms breath-actuated dry powder inhaler |
| c65R. | FLUTICASONE PROPIONATE 250micrograms breath-actuated dry powder inhaler |
| c65S. | FLUTICASONE PROPIONATE 500micrograms breath-actuated dry powder inhaler |
| c65T. | FLIXOTIDE 50micrograms Accuhaler |
| c65U. | FLIXOTIDE 100micrograms Accuhaler |
| c65V. | FLIXOTIDE 250micrograms Accuhaler |
| c65W. | FLIXOTIDE 500micrograms Accuhaler |
| c65X. | FLUTICASONE PROPIONATE 0.5mg/2mL nebulisation units |
| c65Y. | FLUTICASONE PROPIONATE 2mg/2mL nebulisation units |
| c65Z. | FLIXOTIDE 0.5mg/2mL Nebules |
| c65a. | FLIXOTIDE 2mg/2mL Nebules |
| c65b. | FLUTICASONE PROPIONATE 125micrograms CFC-free inhaler |
| c65c. | FLUTICASONE PROPIONATE 250micrograms CFC-free inhaler |
| c65d. | FLIXOTIDE 125micrograms Evohaler |
| c65e. | FLIXOTIDE 250micrograms Evohaler |
| c65f. | FLUTICASONE PROPIONATE 50micrograms CFC-free inhaler |
| c65g. | FLIXOTIDE 50micrograms Evohaler |
| c66.. | BECLOMETASONE DIPROPIONATE [RESPIRATORY USE 2] |
| c661. | *BDP 250micrograms Spacehaler |
| c662. | BECOTIDE 50 EASI-BREATHE inhaler |
| c663. | BECOTIDE 100 EASI-BREATHE inhaler |
| c664. | BECLOFORTE EASI-BREATHE 250micrograms inhaler |
| c665. | QVAR 50 inhaler |
| c666. | QVAR 100 inhaler |
| c667. | QVAR 50 Autohaler |
| c668. | QVAR 100 Autohaler |
| c669. | *BECLAZONE 200 inhaler |
| c66A. | BECLOMETASONE DIPROPIONATE 50micrograms breath-actuated dry powder inhaler |

| Code | Description |
|---|---|
| c66B. | BECLOMETASONE DIPROPIONATE 100micrograms breath-actuated dry powder inhaler |
| c66C. | BECLOMETASONE DIPROPIONATE 250micrograms breath-actuated dry powder inhaler |
| c66D. | ASMABEC 50micrograms Clickhaler |
| c66E. | ASMABEC 100micrograms Clickhaler |
| c66F. | ASMABEC 250micrograms Clickhaler |
| c66G. | BECLOMETASONE DIPROPIONATE 400micrograms breath-actuated dry powder inhaler |
| c66H. | BECLOMETASONE DIPROPIONATE 200micrograms breath-actuated dry powder inhaler |
| c66I. | PULVINAL BECLOMETHASONE DIPROPIONATE 100micrograms breath-actuated dry powder inhaler |
| c66J. | PULVINAL BECLOMETHASONE DIPROPIONATE 200micrograms breath-actuated dry powder inhaler |
| c66K. | PULVINAL BECLOMETHASONE DIPROPIONATE 400micrograms breath-actuated dry powder inhaler |
| c66L. | *BECLOMETASONE 100 cyclocaps |
| c66M. | *BECLOMETASONE 200 cyclocaps |
| c66N. | *BECLOMETASONE 400 cyclocaps |
| c66P. | BECODISK 100micrograms diskhaler 15x8 |
| c66Q. | BECODISK 200micrograms diskhaler 15x8 |
| c66R. | BECODISK 400micrograms diskhaler 15x8 |
| c66S. | BECODISK 100micrograms disk refill 15x8 |
| c66T. | BECODISK 200micrograms disk refill 15x8 |
| c66U. | BECODISK 400micrograms disk refill 15x8 |
| c66V. | BECLOMETASONE DIPROPIONATE 50micrograms CFC-free inhaler |
| c66W. | BECLOMETASONE DIPROPIONATE 100micrograms CFC-free inhaler |
| c66X. | BECLOMETASONE DIPROPIONATE 50micrograms CFC-free breath-actuated aerosol inhaler |
| c66Y. | BECLOMETASONE DIPROPIONATE 100micrograms CFC-free breath-actuated aerosol inhaler |
| c66Z. | QVAR EASI-BREATHE 50micrograms CFC-free breath-actuated dry powder inhaler |
| c66a. | QVAR EASI-BREATHE 100micrograms CFC-free breath-actuated dry powder inhaler |
| c66c. | CLENIL MODULITE 50micrograms CFC-free inhaler |
| c66d. | CLENIL MODULITE 100micrograms CFC-free inhaler |
| c66e. | CLENIL MODULITE 200micrograms CFC-free inhaler |
| c66f. | CLENIL MODULITE 250micrograms CFC-free inhaler |
| c66g. | BECLOMETASONE DIPROPIONATE 200micrograms CFC-free inhaler |
| c66h. | BECLOMETASONE DIPROPIONATE 250micrograms CFC-free inhaler |
| c68.. | MOMETASONE [RESPIRATORY USE] |
| c681. | MOMETASONE FUROATE 200micrograms breath-actuated dry powder inhaler |
| c682. | MOMETASONE FUROATE 400micrograms breath-actuated dry powder inhaler |
| c683. | ASMANEX TWISTHALER 200micrograms breath-actuated dry powder inhaler |
| c684. | ASMANEX TWISTHALER 400micrograms breath-actuated dry powder inhaler |
| c69.. | CICLESONIDE |
| c691. | ALVESCO 160micrograms inhaler |
| c692. | ALVESCO 80micrograms inhaler |
| c69y. | CICLESONIDE 80micrograms inhaler |
| c69z. | CICLESONIDE 160micrograms inhaler |

ICS-LABA

| Code | Description |
|---|---|
| c1D.. | SALMETEROL+FLUTICASONE PROPIONATE |
| c1D1. | SERETIDE 100 Accuhaler |
| c1D2. | SERETIDE 250 Accuhaler |
| c1D3. | SERETIDE 500 Accuhaler |
| c1D4. | SERETIDE 50 Evohaler |
| c1D5. | SERETIDE 125 Evohaler |
| c1D6. | SERETIDE 250 Evohaler |
| c1D7. | SIRDUPLA 25micrograms/125micrograms inhaler |
| c1D8. | SIRDUPLA 25micrograms/250micrograms inhaler |
| c1Du. | SALMETEROL+FLUTICASONE PROPIONATE 25micrograms/50micrograms CFC-free inhaler |
| c1Dv. | SALMETEROL+FLUTICASONE PROPIONATE 25micrograms/125micrograms CFC-free inhaler |
| c1Dw. | SALMETEROL+FLUTICASONE PROPIONATE 25micrograms/250micrograms CFC-free inhaler |
| c1Dx. | SALMETEROL+FLUTICASONE PROPIONATE 50micrograms/100micrograms breath-actuated dry powder inhaler |
| c1Dy. | SALMETEROL+FLUTICASONE PROPIONATE 50micrograms/250micrograms breath-actuated dry powder inhaler |
| c1Dz. | SALMETEROL+FLUTICASONE PROPIONATE 50micrograms/500micrograms breath-actuated dry powder inhaler |
| c1c.. | FLUTICASONE PROPIONATE+FORMOTEROL FUMARATE |
| c1c1. | FLUTIFORM 50micrograms/5micrograms inhaler |
| c1c2. | FLUTIFORM 125micrograms/5micrograms inhaler |
| c1c3. | FLUTIFORM 250micrograms/10micrograms inhaler |
| c1cx. | FLUTICASONE PROPIONATE+FORMOTEROL FUMARATE 250mcg/10mcg inh |
| c1cy. | FLUTICASONE PROPIONATE+FORMOTEROL FUMARATE 125mcg/5mcg inh |
| c1cz. | FLUTICASONE PROPIONATE+FORMOTEROL FUMARATE 50mcg/5mcg inh |
| c67.. | BUDESONIDE+FORMOTEROL |
| c671. | SYMBICORT 100/6 Turbohaler |
| c672. | SYMBICORT 200/6 Turbohaler |
| c673. | SYMBICORT 400/12 Turbohaler |
| c674. | DUORESP SPIROMAX 160mcg/4.5mcg breath-act dry powder inhaler |
| c675. | DUORESP SPIROMAX 320mcg/9mcg breath-act dry powder inhaler |
| c67x. | BUDESONIDE+FORMOTEROL FUMARATE DIHYDRATE 400micrograms/12micrograms breath-actuated dry powder inhaler |
| c67y. | BUDESONIDE+FORMOTEROL FUMARATE DIHYDRATE 200micrograms/6micrograms breath-actuated dry powder inhaler |
| c67z. | BUDESONIDE+FORMOTEROL FUMARATE DIHYDRATE 100micrograms/6micrograms breath-actuated dry powder inhaler |
| c6A.. | BECLOMETASONE+FORMOTEROL |
| c6A1. | FOSTAIR 100micrograms/6micrograms inhaler |
| c6A2. | FOSTAIR NEXTHALER 100micrograms/6micrograms powder inhaler |
| c6Ay. | BECLOMET DIPROP+FORMOTERL FUMARATE DIHYD 100mcg/6mcg pdr inh |
| c6Az. | BECLOMETASONE DIPROPIONATE+FORMETEROL FUMARATE DIHYDRATE 100micrograms/6micrograms inhaler |
| c6B.. | FLUTICASONE+VILANTEROL |
| c6B1. | RELVAR ELLIPTA 184micrograms/22micrograms inhaler |
| c6B2. | FLUTICASONE FUROATE+VILANTEROL 184mcg/22mcg dry pdr inhaler |
| c6B3. | RELVAR ELLIPTA 92micrograms/22micrograms inhaler |
| c6B4. | FLUTICASONE FUROATE+VILANTEROL 92mcg/22mcg dry pdr inhaler |

Theophylline

| Code | Description |
|---|---|
| c41.. | AMINOPHYLLINE |
| c411. | AMINOPHYLLINE 100mg tablets |
| c412. | AMINOPHYLLINE 250mg/10mL injection |
| c413. | AMINOPHYLLINE 500mg/2mL injection |
| c414. | AMINOPHYLLINE 50mg suppositories |
| c415. | AMINOPHYLLINE 100mg suppositories |
| c416. | AMINOPHYLLINE 150mg suppositories |
| c417. | AMINOPHYLLINE 180mg suppositories |
| c418. | AMINOPHYLLINE 360mg suppositories |
| c419. | *THEODROX tablets |
| c41A. | *NORPHYLLIN 100mg tablets |
| c41B. | NORPHYLLIN SR 225mg m/r tablets |
| c41C. | NORPHYLLIN SR 350mg m/r tablets |
| c41a. | PHYLLOCONTIN CONTINUS 225mg m/r tablets |
| c41b. | PHYLLOCONTIN FORTE 350mg m/r tablets |
| c41c. | PHYLLOCONTIN PAEDIATRIC 100mg m/r tablets |
| c41d. | AMINOPHYLLINE 225mg m/r tablets |
| c41e. | *PECRAM 225mg m/r tablets |
| c41f. | AMINOPHYLLINE 350mg m/r tablets |
| c41g. | AMINOPHYLLINE 100mg m/r tablets |
| c41h. | *AMNIVENT 225mg m/r tablets |
| c41i. | *AMNIVENT 350mg m/r tablets |
| c41j. | MIN-I-JET AMINOPHYLLINE 250mg/10mL injection |
| c41k. | AMINOPHYLLINE 250mg/10mL prefilled syringe |
| c41m. | AMINOPHYLLINE HYDRATE 225mg m/r tablets |
| c43.. | THEOPHYLLINE |
| c431. | *BIOPHYLLINE 125mg/5mL syrup |
| c432. | *NUELIN 125mg tablets |
| c433. | *NUELIN 60mg/5mL liquid |
| c434. | *LASMA 300mg m/r tablets |
| c435. | NUELIN SA 175mg m/r tablets |
| c436. | NUELIN SA-250 250mg m/r tablets |
| c437. | *PRO-VENT 300mg m/r capsules |
| c438. | SLO-PHYLLIN 60mg m/r capsules |
| c439. | SLO-PHYLLIN 125mg m/r capsules |
| c43A. | THEOPHYLLINE 200mg/10mL injection |
| c43B. | THEOPHYLLINE 10mg/5mL sugar free solution |
| c43a. | SLO-PHYLLIN 250mg m/r capsules |
| c43b. | *THEO-DUR 200mg m/r tablets |
| c43c. | *THEO-DUR 300mg m/r tablets |
| c43d. | *THEOGRAD 350mg m/r tablets |
| c43e. | UNIPHYLLIN CONTINUS 400mg m/r tablets |
| c43f. | UNIPHYLLIN CONTINUS 200mg m/r tablets |
| c43g. | LABOPHYLLINE 200mg/10mL injection |
| c43h. | UNIPHYLLIN CONTINUS 300mg m/r tablets |
| c43i. | *BIOPHYLLINE 350mg m/r tablets |
| c43j. | *BIOPHYLLINE 500mg m/r tablets |
| c43k. | THEOPHYLLINE 500mg m/r tablets |
| c43m. | *THEOPHYLLINE 125mg/5mL syrup |
| c43n. | *THEOPHYLLINE 125mg tablets |
| c43o. | *THEOPHYLLINE 60mg/5mL liquid |
| c43p. | THEOPHYLLINE 175mg m/r tablets |
| c43q. | THEOPHYLLINE 250mg m/r tablets |
| c43r. | THEOPHYLLINE 300mg m/r capsules |
| c43s. | THEOPHYLLINE 60mg m/r capsules |
| c43t. | THEOPHYLLINE 125mg m/r capsules |

| Code | Description |
|---|---|
| c43u. | THEOPHYLLINE 250mg m/r capsules |
| c43v. | THEOPHYLLINE 200mg m/r tablets |
| c43w. | THEOPHYLLINE 300mg m/r tablets |
| c43x. | THEOPHYLLINE 350mg m/r tablets |
| c43y. | THEOPHYLLINE 400mg m/r tablets |
| c43z. | *THEOPHYLLINE 200mg tablets |
| Leukotriene receptor antagonist (LTRA) | |
| cA... | LEUKOTRIENE RECEPTOR ANTAGONIST |
| cA1.. | MONTELUKAST |
| cA11. | MONTELUKAST 10mg tablets |
| cA12. | MONTELUKAST 5mg chewable tablets |
| cA13. | SINGULAIR 10mg tablets |
| cA14. | SINGULAIR PAEDIATRIC 5mg chewable tablets |
| cA15. | SINGULAIR PAEDIATRIC 4mg chewable tablets |
| cA16. | SINGULAIR PAEDIATRIC 4mg/sachet granules |
| cA1y. | MONTELUKAST 4mg/sachet granules |
| cA1z. | MONTELUKAST 4mg chewable tablets |
| cA2.. | ZAFIRLUKAST |
| cA21. | ZAFIRLUKAST 20mg tablets |
| cA22. | ACCOLATE 20mg tablets |
| Sodium cromoglicate | |
| c71.. | SODIUM CROMOGLICATE [ASTHMA] |
| c711. | *INTAL 1mg inhaler |
| c712. | *INTAL HALERMATIC insufflator |
| c713. | *INTAL 20mg spincaps |
| c714. | *INTAL SPINHALER insufflator |
| c715. | INTAL 20mg/2mL nebuliser solution |
| c716. | *INTAL 5mg inhaler |
| c717. | SODIUM CROMOGLICATE 20mg inhalation capsules |
| c718. | SODIUM CROMOGLICATE 20mg/2mL nebuliser solution |
| c719. | SODIUM CROMOGLICATE 5mg inhaler |
| c71a. | *INTAL 5mg Autohaler |
| c71b. | STERI-NEB CROMOGEN 20mg nebulisation units |
| c71c. | *CROMOGEN 5mg inhaler |
| c71d. | INTAL FISONAIR 5mg inhaler + spacer device |
| c71e. | SODIUM CROMOGLICATE 5mg inhaler + spacer device |
| c71f. | SODIUM CROMOGLYCATE 5mg auto inhaler |
| c71g. | INTAL SYNCRONER 5mg inhaler + spacer device 2x112dose |
| c71h. | SODIUM CROMOGLICATE 5mg breath-actuated aerosol inhaler |
| c71i. | INTAL 5mg CFC-free inhaler |
| c71j. | CROMOGEN EASI-BREATHE 5mg breath-actuated aerosol inhaler |
| c71k. | SODIUM CROMOGLICATE 5mg CFC-free inhaler |
| c72.. | SODIUM CROMOGLICATE COMPOUNDS |
| c721. | *INTAL COMPOUND spincaps |
| c722. | *AEROCROM inhaler |
| c723. | AEROCROM SYNCRONER inhaler + spacer device |
| c72y. | SODIUM CROMOGLICATE+SALBUTAMOL 1mg/100micrograms inhaler + spacer device |
| c72z. | SODIUM CROMOGLICATE+SALBUTAMOL 1mg/100micrograms inhaler |
| Nedocromil | |
| c74.. | NEDOCROMIL SODIUM [ASTHMA] |
| c741. | *TILADE MINT 2mg inhaler |
| c742. | *NEDOCROMIL SODIUM 2mg inhaler |
| c743. | *TILADE MINT 2mg inhaler |
| c744. | TILADE MINT SYNCRONER 2mg inhaler |
| c745. | NEDOCROMIL SODIUM 2mg inhaler + spacer |
| c746. | NEDOCROMIL SODIUM 2mg CFC-free inhaler |
| c747. | TILADE 2mg CFC-free inhaler |
| Oral corticosteroids | |
| fe61 | PREDNISOLONE 1mg tablets |
| fe62 | PREDNISOLONE 5mg tablets |
| fe66 | DELTACORTRIL ENTERIC 5mg tablets |
| fe6i | PREDNISOLONE 5mg e/c tablets |
| fe6j | PREDNISOLONE 5mg soluble tablets |
| fe6k | PREDNISOLONE 50mg tablets |
| fe6z | PREDNISOLONE 25mg tablets |
