# Supplementary material for: Association of socioeconomic deprivation with asthma care, outcomes, and deaths in Wales: A 5-year national linked primary and secondary care cohort study
Source: PLoS Med. 2021 Feb 12;18(2):e1003497. doi: 10.1371/journal.pmed.1003497 (PMC7880491; doi:10.1371/journal.pmed.1003497)

S1 Fig: Distribution of the Welsh Index of Multiple Deprivation (WIMD) 2011 score and its quintiles in the study cohort

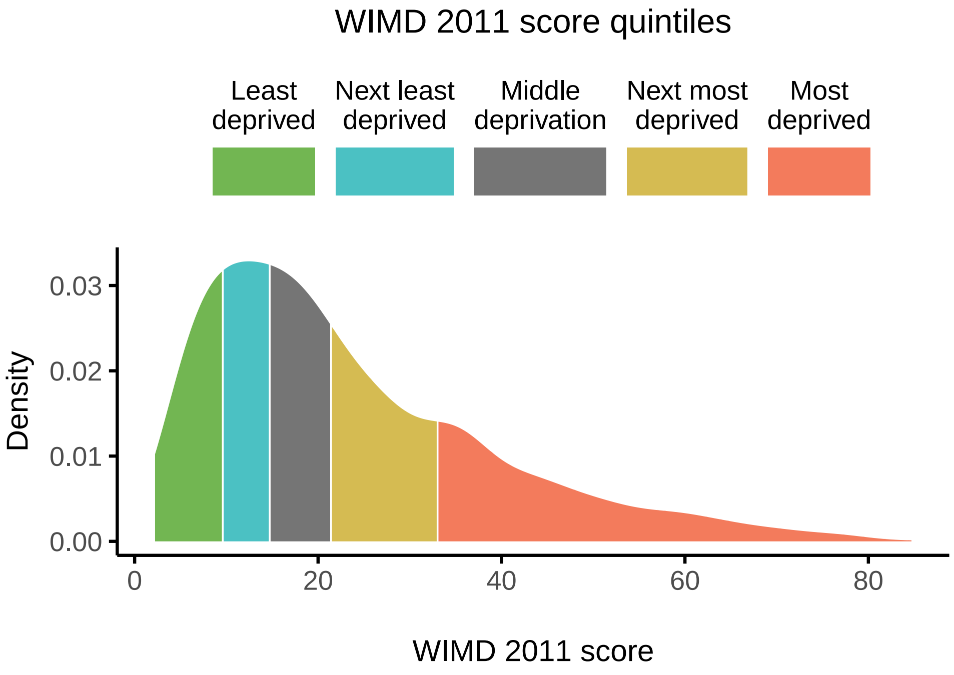

Supplement: S1 Fig — WIMD, Welsh Index of Multiple Deprivation. (PDF) [file pmed.1003497.s006.pdf]
