# Supplementary material for: Association of socioeconomic deprivation with asthma care, outcomes, and deaths in Wales: A 5-year national linked primary and secondary care cohort study
Source: PLoS Med. 2021 Feb 12;18(2):e1003497. doi: 10.1371/journal.pmed.1003497 (PMC7880491; doi:10.1371/journal.pmed.1003497)
Supplement: S2 Fig — NB, negative binomial. (PDF) [file pmed.1003497.s007.pdf]

S2 Fig: Model fit for the negative binomial generalised linear regression models

(a)

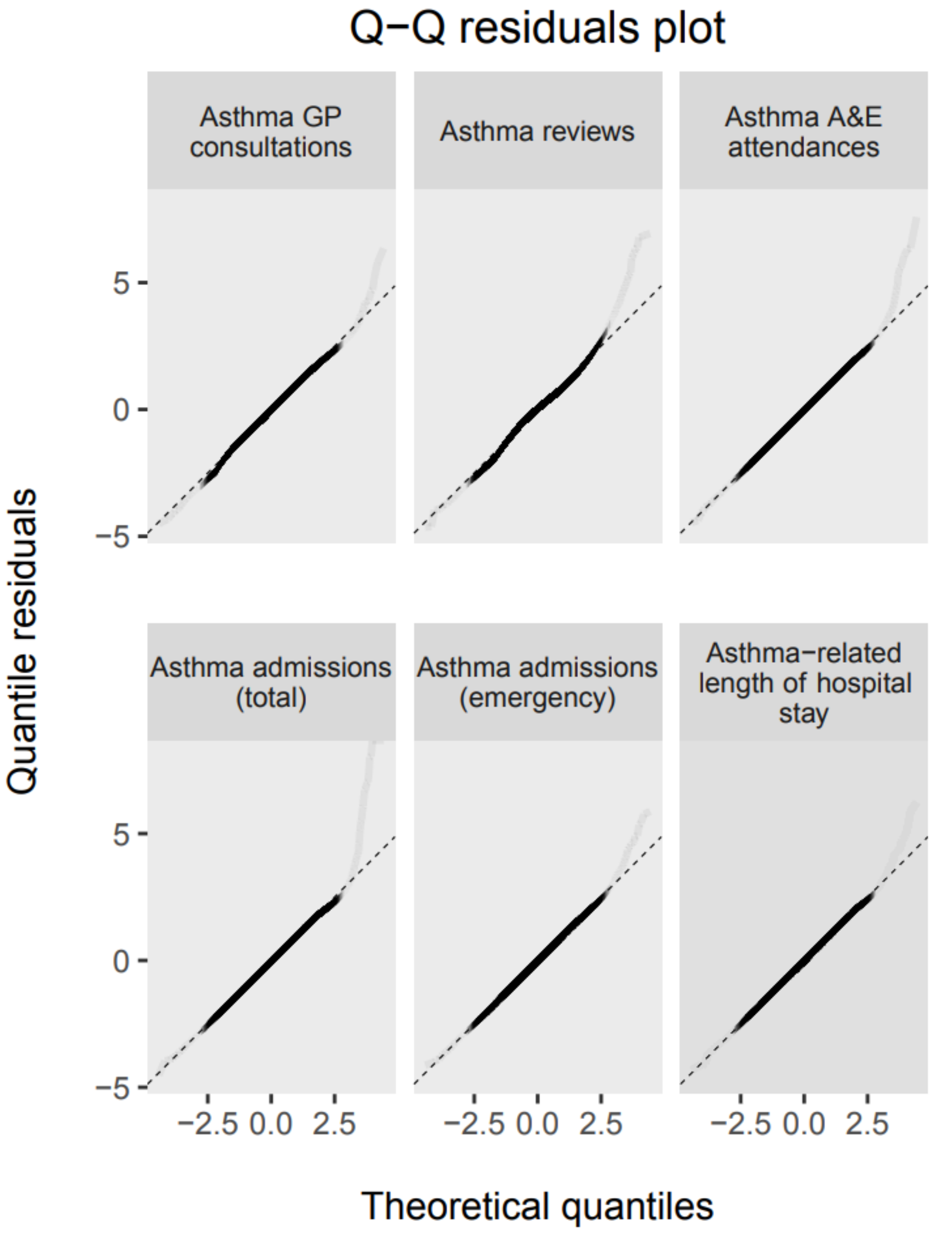

Quantile-quantile plot for the model residuals. Line colour represents the density of individual points. The raw residuals in the NB models followed a normal distribution, except for right skewness in the asthma reviews and length of stay models. A&E = accident and emergency; GP = general practitioner.

(b)

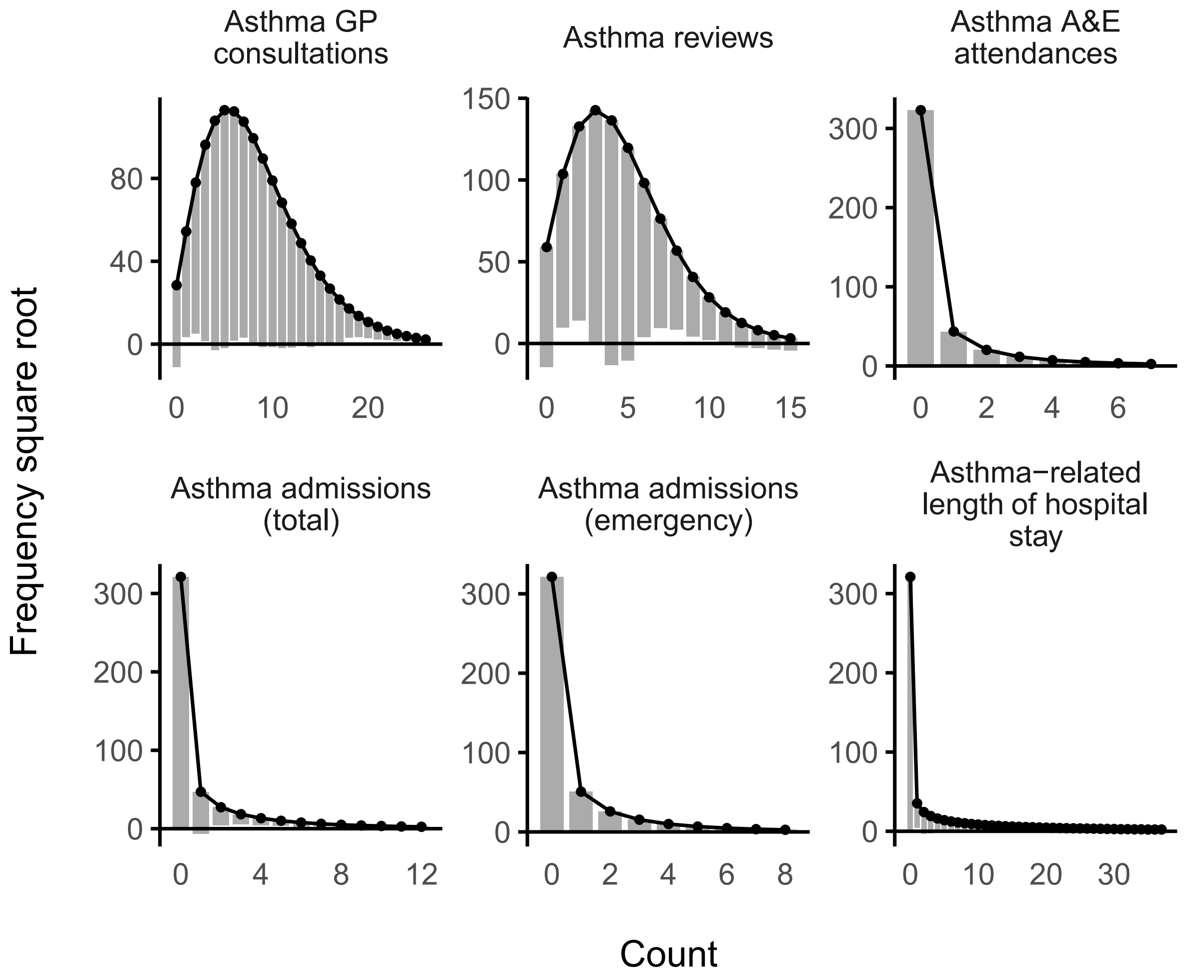

Rootograms for the negative binomial models of the count variables. The rootograms demonstrated that the deviations between the observed and predicted counts were relatively small, with more deviations in the asthma-related GP consultations and review models. A&E = accident and emergency; GP = general practitioner.
