# Supplementary material for: Association of socioeconomic deprivation with asthma care, outcomes, and deaths in Wales: A 5-year national linked primary and secondary care cohort study
Source: PLoS Med. 2021 Feb 12;18(2):e1003497. doi: 10.1371/journal.pmed.1003497 (PMC7880491; doi:10.1371/journal.pmed.1003497)

S3 Fig: Distribution of Asthma Medication Ratio (AMR) across the Welsh Index of Multiple Deprivation 2011 quintiles

AMR was calculated using the formula (ICS + ICS_LABA + sodium cromoglicate + nedocromil)/(ICS + ICS_LABA + sodium cromoglicate + nedocromil + SABA) using the 5-year counts.ICS = inhaled corticosteroid; LABA = long-acting beta adrenoceptor agonist; SABA = short-acting beta agonist.

(a)

The density distributions (below) demonstrate lower AMRs in the more deprived quintiles. The blue lines represent the means within WIMD quintiles, while the dotted line represents the overall mean.

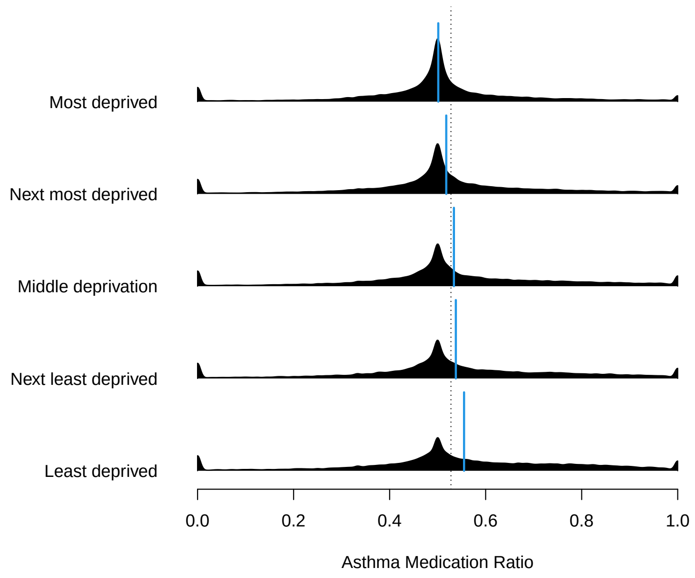

(b)

The cumulative distributions of AMR (below) under the value of 0.5 were similar across the WIMD quintiles. However, in the more deprived quintiles there were fewer patients with AMR > 0.5.

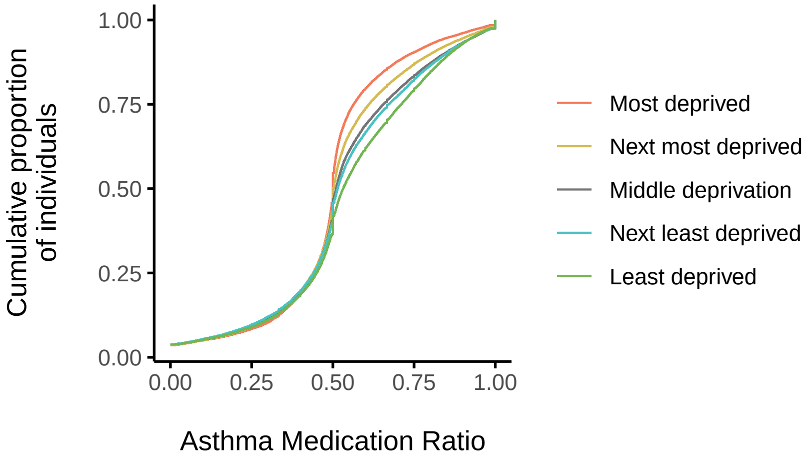

Supplement: S3 Fig — AMR, asthma medication ratio; WIMD, Welsh Index of Multiple Deprivation. (PDF) [file pmed.1003497.s008.pdf]
