# Supplementary material for: Association of socioeconomic deprivation with asthma care, outcomes, and deaths in Wales: A 5-year national linked primary and secondary care cohort study
Source: PLoS Med. 2021 Feb 12;18(2):e1003497. doi: 10.1371/journal.pmed.1003497 (PMC7880491; doi:10.1371/journal.pmed.1003497)
Supplement: S4 Fig — WIMD, Welsh Index of Multiple Deprivation. (PDF) [file pmed.1003497.s009.pdf]

S4 Fig: Proportion of emergency-to-total admissions in each quintile of the Welsh Index of Multiple Deprivation (WIMD) 2011 in the study cohort

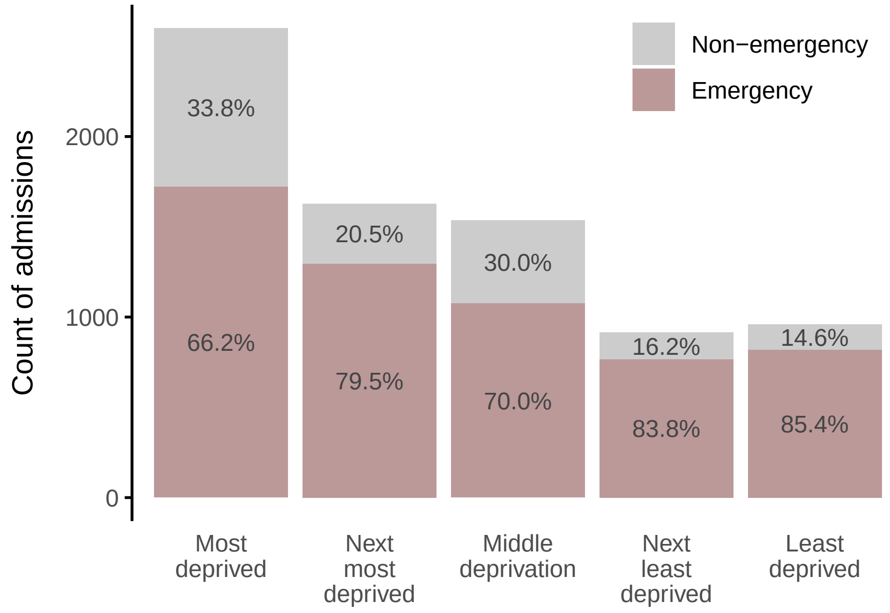

Proportion of emergency-to-total admissions in each quintile of the Welsh Index of Multiple Deprivation (WIMD) 2011 in the study cohort. Although the most deprived areas had the highest rate of asthma admissions, these areas had the highest proportion of non-emergency to total asthma admissions.
