# Supplementary material for: Association of socioeconomic deprivation with asthma care, outcomes, and deaths in Wales: A 5-year national linked primary and secondary care cohort study
Source: PLoS Med. 2021 Feb 12;18(2):e1003497. doi: 10.1371/journal.pmed.1003497 (PMC7880491; doi:10.1371/journal.pmed.1003497)
Supplement: Attachment — Submitted filename: rebuttal_letter.pdf [file pmed.1003497.s010.pdf]

Rebuttal letter

PMEDICINE-D-20-01743R1

Association of socioeconomic deprivation with asthma care, outcomes, and deaths in Wales: a five-year national linked primary and secondary care cohort study

---

We would like to thank the reviewers and editors for their thoughtful comments and efforts towards improving our manuscript. Please find below our point-by-point responses.

Reviewer 1

I confine my remarks to statistical aspects of this paper.

I have several issues to resolve before I can recommend publication.

> **1.** First, in many places, the authors use causal language (e.g. "impact") but this is not warranted. Words such as "associated" should be used instead.

Thank you for your helpful suggestion. We agree and we have now carefully checked the entire manuscript and revised text where necessary to ensure causal language is carefully used.

For example, we have changed the title to:

> ~~Impact~~ Association of socioeconomic deprivation with asthma care and outcomes in Wales ...

> **2.** Second, using quantiles of the wealth index is not good. In *Regression Modelling Strategies* Frank Harrell lists 11 problems with this and sums up "nothing could be more disastrous". Leave the index as a number and use splines to investigate nonlinearities. This will change some of the graphs (e.g. you can use density plots of index).

Many thanks for your comment.

We agree on the pitfalls of categorisation. Therefore, in response to your request, we have additionally presented generalised additive models (GAMs) of the outcomes against the numerical score of the Welsh Index of Multiple Deprivation (WIMD) 2011. We used Simon Woods's R package `mgcv` for this purpose. Results are presented as plots of the smooths that represent the effect of WIMD's numerical score (and age) on the outcomes. These GAMs are consistent with most of the quintile-based generalized linear models, and therefore we are presenting both approaches.

> **3.** Line 104ff I think that a beta regression would be better here. Logistic regression is used when the outcome is categorical. Here, it is continuous but bounded.

Many thanks for this helpful comment.

In the original submission, logistic regression was only used to model asthma mortality, a dichotomous categorical variable and not a bounded continuous variable.

We think the statistical reviewer refers to Asthma Medication Ratio (AMR) which ranges from 0 to 1. We have now modelled AMR in a generalised additive model using the `mgcv::betar` (beta regression) family.

> **4.** line 127 Why not use interactions with sex?
>
> Peter Flom

Many thanks for this suggestion. We agree with it.

We have now added binomial generalized additive models for asthma deaths.

We modelled deaths against the deprivation score within each sex and calculated a difference smooth to explore the between-sex variation in the deprivation score effect (please see Lines 180-186 and Fig 13).

Reviewer 2

> **5.** The AMR was calculated by counting the number of ICS, ICS-LABA and SABA prescriptions. While this approach has the virtue of simplicity a drawback is that inhalers with different strengths or different numbers of doses are counted the same. Can the authors justify why they took this approach rather than counting the number of doses, or better still by calculating the total quantity of inhaled steroid used (in beclomethasone dipropionate equivalent units) and comparing this to the number of reliever doses?

Thank you for your comment.

We agree with the point you are making. However, one of the limitations of our system is that we do not have data on the number of doses (puffs per day). However, the Asthma Medication Ratio (AMR) formula has already been validated to in the United States against a number of validated quality of life measures and healthcare utilisation records [1]. This formula was based on the number of medications rather than strengths or doses.

We have added the lack of data on actual doses to the section on limitations.

> The AMR formula is based on the number of prescriptions which did not necessarily reflect the actual prescribed dosage (puffs per day). However, data on actual dosage are currently not available in the primary care dataset that we have used.

6. Some of the data in Table 2 appear to be incorrect eg. Numbers of SABA, ICS and ICS-LABA inhalers seem too large and do not tally with the text. Also the number of A&E visits does not seem to tally between Table 2 and the text (line 185). Please could the authors carefully check all data in the text and tables to ensure they are correct.

Thank you for this comment. We have checked the data. Table 2 was not incompatible with the text but they were describing two different metrics on the same construct. In Table 2, the numbers of SABA, ICS and ICS-LABA inhalers (and other variables) were for the 5-year follow-up period whereas in the text they were reported per year. For example, people in the most deprived quintile had an average of 36.7 inhalers over 5 years, which corresponds to an annual number of 7.34 (rounded as 7.3). However, for consistency, Table 2 now includes the average per year of these events rather the 5-year total.

In the text, the yearly mean of ICS + ICS_LABA per WIMD quintile incorrectly included other categories such as oral corticosteroids and theophylline. We replaced this number with the total of ICS, ICS-LABA, sodium cromoglicate, and/or nedocromil (which is 7.0 and 5.4 for the most and least deprived quintiles).

Table 2 presents observed counts. However, the A&E visits and length of hospital stay (LOS) that were reported in the text were predicted from the models. We have removed them to avoid confusion with Table 2.

7. A number of variables in Table 2 are heavily skewed to the right (eg. Length of stay, A&E visits, hospitalisations) so should not be expressed as mean (SD).

Many thanks for this comment. In Table 2, we have replaced *mean (SD)* with *mean, median (Q1-Q3)*.

8. It is surprising that approximately 30% of hospital admissions were deemed as 'elective'. Elective admissions for asthma are very rare and I would expect 99% of admissions to be emergencies. This may be a coding issue and I would suggest simply presenting data on hospital admissions in aggregate.

Thank you for raising this point and for your suggestion.

Our focus was on emergency admissions which accounted for 70% of all 'admissions' in the study cohort. We have double-checked our database script to extract admission type and it was correct. We have used the official list of Admission Method codes to identify emergency admissions from the PEDW dataset which is published on NHS

Wales Informatics Service (NWIS) website [2]. Therefore, our definition of emergency admissions represents the official definition and is meant to be interpreted as such.

The majority (58.6%) of the 'non-emergency' admissions were coded as day-case admissions (e.g., to receive biologics). A Respiratory Physician (GAD) and co-authors has checked these numbers and they corroborate with the expected number of asthma patients being admitted as day cases for biologic therapies across Wales in the last decade.

Although our focus was on emergency admissions, we believe that by presenting both admission types (total and 'emergency') we present a e complete picture about asthma admissions.

9. It should be pointed out in the discussion that, while possibly reaching statistical significance, the difference in GP visits and asthma reviews between the lowest and highest deprivation quintiles is negligible and probably not of clinical significance.

Thank you for this comment.

We agree the difference is very small and has probably no clinical significance, and we have amended the Discussion accordingly.

> The socioeconomic variation in asthma-related primary care consultations and structured asthma review is marginal and may have no clinical significance. However, the wider gap in emergency asthma care and deaths indicates inequalities in disease severity and potentially in how the disease is managed.

10. Line 285-6: The authors mention that they did not perform sub-analyses for age groups - however, they have the data to do this and it would be useful to see if they could replicate the rather counter-intuitive results seen in England by Gupta et al.

Thank you for the suggestion.

We have now explored the interaction between the Welsh Index of Multiple Deprivation (WIMD) 2011 score and age. However, we did not find the unexpected pattern of decreasing asthma mortality in more deprived areas among the 5-44year-olds which was highlighted by Gupta et al. [3]. Interestingly, we found it among the over-75-year-old males (please refer to Fig 13).

11. What does the IRR in Line 199 refer to? I am unclear what incidence is being referred to in the context of the length of stay analysis (see also in Table 3).

We treated length of hospital stay (LOS) as a count variable (count of days) rather than a continuous variable. Therefore, we used count models for this variable, from which incidence rate ratios can be estimated. The incidence rate ratio (IRR) refers to the relative

risk of incurring an additional hospital day compared with the least deprived quintile (the reference).

We have added the following clarification to the Methods:

> We treated LOS in hospital as a count variable (count of days) for which the model coefficient represents the incidence rate ratio (IRR) of incurring an additional day in hospital in a given quintile compared with the least deprived quintile.

Reporting LOS using IRR has been used elsewhere [4, 5].

12. The manuscript is generally well written, but there were a few typos I picked up: Line 56: Should read "Figure 1 illustrates..."; Line 88: There appear to be some missing words.; Line 116: Should read "For each count outcome variable..."

We apologise for these. We have now corrected these typos.

Reviewer 3

13. A major weakness of the study is that the analysis does not control for individual-/household-level socioeconomic status (SES). Consequently, the area-based measure of socioeconomic deprivation is most likely confounded by individual-/household SES, and the interpretation of the study's findings is less clear from that perspective. For example, a IRR of 1.58 for emergency admissions for asthma is observed in the most deprived quintile vs. least deprived quintile. This would appear to be an important association, except for the fact that individual-/household SES most likely accounts for much of this association. In that event, what is the value/implication of identifying a IRR of 1.58?

We agree about the limitations of an area-based deprivation index as a proxy for individual socioeconomic deprivation. However, person-level socioeconomic deprivation data, e.g., income data from the Tax Department or benefits income from Department for Work and Pensions (DWP) or educational data from Census, were not available in our data linkage project and such linkage is out of scope of this study.

However, we have revised the text to ensure that our findings primarily describe areas rather than individuals (e.g., people living in the most deprived areas were more likely to have emergency admission for asthma) and added the following to the limitations:

> The WIMD 2011 is an area-level index [6] and therefore caution is required when drawing person-level inferences.

We believe an IRR of 1.58 at the area level—i.e. the most deprived areas have seen 58% more asthma emergency admissions per asthma patient—is large enough to warrant intervention by health service providers and health policy makers.

14. It is unclear why the outcome of asthma-related death is used. This refers to any mention of asthma in the death record, but not necessarily asthma as the underlying cause of death. Such a lack of specificity is a disadvantage in my opinion.

Thank you for this comment. Identifying asthma deaths using any mention on the death record is a position also taken by the UK Office of National Statistics [2]:

> *For asthma, diabetes mellitus (types 1 and 2) and sepsis, deaths have been included where the condition is mentioned on the death certificate and where it is the underlying cause, as these conditions will often contribute to a death occurring but not being recorded as the underlying cause.*

Building on your suggestion, we have now also analysed both definitions: death with any mention of asthma, and death with asthma as the underlying cause. We believe that taking both the wider and the strict definitions into account is likely to provide the most meaningful analysis of asthma-related deaths, since the number of deaths with asthma as the underlying cause is small.

15. 15. Some description is needed about the relevance of the area units used to measure socioeconomic deprivation (LSOAs). In addition, the authors state that they used the address with the longest duration during the follow-up period (2013-2017). What assumptions are being made about the timing of exposure in the LSOA in relation to the asthma outcomes – is such a short-term effect really plausible and supported empirically in previous studies?

The use of LSOAs as area units to measure socioeconomic deprivation was the approach taken by the Welsh Government, advocated by the Office of National Statistics. We added the following text about LSOAs:

> The WIMD 2011 is based on Lower Layer Super Output Areas (LSOAs) which is a small-area geography designed by the UK Office for National Statistics (ONS) for census-related purposes with consistent population sizes (1,500 people on average) [6].

To our knowledge, there is no standard approach of adjusting for change in socioeconomic deprivation during the follow-up period due to change in address. However, we believe that choosing the Welsh Index of Multiple Deprivation (WIMD) score (and its quintile) as that of the address of the longest duration (within the study period) is reasonable as this reflects the greatest period of exposure to the socioeconomic circumstances of living in such an area. Indeed, only 17.6% of the study cohort changed addresses during the study period. The mean change in the overall WIMD 2011 score between the patients longest address and other addresses was 0.2 (SD = 17.2), which is small compared with the range of this score in the study population ($\sim$2 to $\sim$84) and therefore it is unlikely to affect the findings.

16. The authors cite smoking as a potential residual confounder. However, it seems infeasible that smoking (as a confounder) would cause area-level socioeconomic deprivation. What other residual confounders were unable to be controlled for in the analysis that can be commented on in the Discussion?

Smoking was suggested as a confounder to the direct association between the WIMD quintile and asthma-related outcomes (e.g., A&E attendances and emergency admissions for asthma). The potential confounding effect of smoking, regardless of its magnitude, may exist since it (1) increases the risk of a number of chronic diseases that are included in the health domain of the WIMD 2011 index, and (2) can worsen asthma.

We now also discuss comorbidities as potential residual confounders:

> Asthma is also associated with a range of comorbidities, such as obesity and depression, which are associated with higher asthma severity and lower control and in which a socio-economic gradient has been observed [7, 8].

17. The study cohort consisted of individuals with ever-diagnosed, continuously-treated asthma. If those who are socioeconomically deprived are less likely to be diagnosed and/or continuously treated for asthma, how might that have affected the findings of the study and their interpretation?

Thank you for this insightful comment.

For those with undiagnosed asthma, the answer requires a different analysis of data that is out of the scope of this study.

As shown in the original submission, people with no continuous treatment (i.e. did not satisfy the condition of at least one prescription per year) were included, along with the continuously treated, in a sensitivity analysis (in the supplementary materials) in which the findings did not significantly differ from those in the main cohort (i.e. the inequality gap still exists).

18. For the negative binomial model that was applied, please clarify what offset was used, and please indicate any evidence that the NB model was more appropriate than a zero-inflated NB model.

We have not used an offset in the negative binomial models as all the study individuals in the main cohort had the same follow-up period.

The ratio of observed to expected zeros in ED attendances and admissions was almost 1 but was 1.6 and 1.8 for asthma GP consultations and reviews. However, an earlier analysis presented in the doctoral thesis of the first author found very similar IRRs using zero-inflated negative binomial models (although these were based on an earlier follow-up period 2010-2014) [9].

Comments from the academic editor

19. I think reviewer 3's biggest issue around confounding individual/household wealth and other factors such as smoking vs geographic deprivation is an important one but it is simply not something that can be addressed with this sort of data linkage study. It would require linkage to other datasets (e.g census data) and that seems out of scope for this study.

Thank you for this important point. We agree and make this point in our response to Reviewer 3 (comment 13).

Other key points:

20. The effect sizes are really very small for primary care utilisation and asthma reviews and although statistically significant I am not sure they translate to anything meaningful. Also for primary care and deaths, there does not seem much difference across quintiles 1-4 and only a difference with quintile 5. This suggests the association is not that clear when using categorical measures of deprivation and relates to reviewer 1's point about looking at it by numerical index.

Thank you for this point.

We agree that the socioeconomic variations in GP consultations and reviews are very small, and we have amended the Discussion accordingly.

> The socioeconomic variation in asthma-related primary care consultations and structured asthma review is marginal and may have no clinical significance. However, the wider gap in emergency asthma care and deaths indicates inequalities in disease severity and potentially in how the disease is managed.

On the latter point, we have now explored the non-linear associations between the deprivation score and each of our study outcomes in response to Reviewer 1's comment #2, as shown in the figures 2 to 14.

21. I agree with reviewer 2 on many points and won't repeat them here except to emphasise the issue of defining medication use and converting to beclomethasone equivalents would make for more robust analyses. On Table 2, despite higher use of ICS in most deprived regions overall, the AMR is lower which perplexes me a little. This suggests it is a dosing/ adherence issue rather than prescribing in the first place.

Thank you for your comment. Please see our response to Reviewers 2's comment (#5) regarding the use of prescription counts rather than strength and dosage.

Regarding lower AMR in the most deprived areas despite higher ICS use:

AMR, as a prescribing quality measure, is concerned with the balance between controller and reliever prescriptions regardless of their absolute counts. Schatz et al. [1, 10] showed that controller and reliever counts corresponded to disease severity, whereas AMR was better as a quality of care indicator. Schatz et al. [11] also wrote:

> *The number of SABAs dispensed is even more strongly related to subsequent asthma exacerbations than the medication ratio measure. However, the medication ratio measure has one advantage over SABA dispensings as a quality-of-care marker. The SABA measure does not account for severity or the attempt to appropriately use controllers. A patient with severe treated disease might still require high levels of SABAs, but a high medication ratio at least reflects an attempt to use controllers to manage the illness. This would further support the use of the medication ratio measure as an administrative data asthma quality-of-care marker.*

In our study, the higher ICS use in the most deprived areas may potentially be explained, at least partly, by a more severe and/or less controlled disease which consequently requires higher use of medications. Adherence could play a role, as does the dosing. Dispensing data would allow exploring these factors, but the research project does not yet have access to these data.

We have updated the Discussion to reflect this point.

> The modestly higher use of both controller and reliever prescriptions with higher overall deprivation is consistent with a gradient in asthma severity and/or control. However, the corresponding inverse gradient of lower controller-to-total medication ratio—a measure of whether controllers are adequate relative to relievers—with higher deprivation suggests variations in prescribing, dosing, adherence, and/or asthma self-management [12].

22. Although the authors did a sensitivity analysis on people with non-continuous follow-up, I would have liked to see a sensitivity analysis that was not predicated on having at least one asthma prescription. There are a lot of people from Figure 2 who are excluded on this criterion. This group (non-prescribed) is likely heterogeneous and could influence the results in either direction - they have mild asthma and therefore don't use any services (primary care or hospital) or they have more severe asthma and don't access any treatment and get worse outcomes.

We would like to clarify how we performed the sensitivity analysis in relation to selection criteria. In the previous version, as Figure 2 showed (S2 in the revised manuscript), the main study cohort included those with **(1)** continuous follow-up in the primary care dataset and also **(2)** with at least one asthma prescription every year.

By dropping **(1)**, we included not only people with continuous follow-up but also those with gaps in their follow-up. By dropping **(2)**, we effectively included people with any number, including zero, prescriptions during the follow-up. Therefore, the non-prescribed people were already included in this sensitivity analysis. The results of the sensitivity

analyses were presented in the supplementary materials.

We have amended the following sentences about the sensitivity analyses in the Methods to make it clearer:

> However, we performed two separate sensitivity analyses; in the first one we removed the condition of having continuous asthma treatment over the follow-up period, and in the second one we removed the condition of continuous follow-up in the primary care dataset.

> **23.** I think all primary results should be gender disaggregated given the findings from Table 4 and 5. I would be keen for more understanding to explain the much higher female utilisation of ED and hospitalisation overall. How does this relate to the current literature on the topic. Also it should be stated in the limitations that females were over-represented in primary health care datasets (this is a well-known limitation).

Thank you for this suggestion.

We have done this in the new analysis (generalised additive models) including estimating the effect of WIMD score within each gender, and also the variation of this effect between males and females. We also present the overall predicted healthcare resource utilisation counts and risk for deaths for each gender across the WIMD score.

Even though females are generally overrepresented in UK primary care, the gender differences in asthma-related primary care use is minimal (IRR = 1.03). The gap is much wider in the recorded asthma emergency care and deaths, which could be explained by the well-known gender differences in asthma development and outcomes.

We have reviewed the current literature and added the following to the Discussion:

> Females, especially between the age of 16 and 60 years, are generally over-represented in primary care [13]. Nonetheless, in our study, the gender gap in asthma-related primary care consultations was minimal. However, females had higher rates of asthma-related accident and emergency (A&E) attendances, admissions in middle age and higher risk of asthma deaths among all adults, with the inverse pattern in childhood, which is generally consistent with other studies [14, 15, 16, 17, 18]. These patterns could be driven by gender differences in asthma development, disease experience, and outcomes [19, 20].

> **24.** The authors have not done any age sub-group analyses and I would be keen to understand to what extent childhood vs adult asthma is driving the utilisation patterns observed.

Thank you for this suggestion.

We have done this in the new analysis (generalised additive models) which includes

patterns of the effect of age on the health resource utilisation, we found minimal age variations in asthma primary care, with younger age having slightly more GP consultations for asthma, and middle age having slightly fewer asthma reviews. The variations were greater in accident and emergency attendances and emergency admissions, where the younger people had higher rates (please refer to Fig 4). Middle age had the lowest Asthma Medication Ratios (please refer to Fig 6).

We also included full tensor smooths illustrating the interaction between age and the WIMD score, which shows varying patterns across the outcome variables (please refer to Fig 8).

> **25.** The discussion suggests that more needs to be done in primary care for the most deprived groups but I think there needs to be much greater emphasis on social determinants of health and going outside the health care system to reduce inequities. I am fairly sceptical that improving primary health care would do much to address the disparities identified in this paper.

We agree with this general point and have updated the Discussion regarding the pressing need to achieve equitable wider social determinants of health.

> However, those interventions alone are unlikely to bridge the socioeconomic gap in asthma outcomes. Rather, structural and social determinants, including the circumstances in which people are born and live, play a crucial role in asthma outcomes [21]. As with health inequalities in general, inequalities in asthma should ultimately be addressed by achieving equitable wider societal determinants of health, particularly educational opportunities, housing, and health service resourcing [22].

However, we still consider that primary care can contribute more to address asthma-specific socioeconomic variations, such as that in the reliever-to-total medication ratio by ensuring adequate prescribing of controllers, emphasizing their importance to patients, improving their asthma education, and supporting asthma self-management.

Requests from the editors

> **26.** *As noted by one reviewer, it would be important to be careful around use of causal language given the study design, and there is not always consistency throughout the paper.

Thank you. We have carefully checked the entire manuscript and revised text where necessary to ensure causal language is carefully used.

27. Please structure your abstract using the PLOS Medicine headings (Background, Methods and Findings, Conclusions - "Methods and Findings" should be a single subsection header).

Thank you. We have restructured the abstract as requested.

28. In the last sentence of the Abstract Methods and Findings section, please include a brief summary of any key limitation(s) of the study's methodology.

Thank you. We have added key limitations at the end of Abstract Methods and Findings section.

29. If possible, please reformat the intext reference callouts into PLOS Medicine style (sequential numerals in square brackets) - if using referencing software this should be fairly straight forward.

Many thanks

Thank you. We have formatted the intext reference callouts according to PLOS Medicine style.

30. At this stage, we ask that you include a short, non-technical Author Summary of your research to make findings accessible to a wide audience that includes both scientists and non-scientists. The Author Summary should immediately follow the Abstract in your revised manuscript. This text is subject to editorial change and should be distinct from the scientific abstract. Please see our author guidelines for more information: https://journals.plos.org/plosmedicine/ s/revising-your-manuscript#loc-author-summary

Thank you. We have now included an Author Summary.

31. Did your study have a prospective protocol or analysis plan? Please state this (either way) early in the Methods section.

a) Please clarify whether the analytical approach followed in this paper corresponded to one laid out in a prospective analysis plan (from your funding proposal, IRB or other ethics committee submission, study protocol, or other planning document written before analyzing the data) was used in designing the study, please include the relevant prospectively written document with your revised manuscript as a Supporting Information file to be published alongside your study, and cite it in the Methods section. A legend for this file should be included at the end of your manuscript.

b) If no such document exists, please make sure that the Methods section transparently describes when analyses were planned, and when/why any data-driven changes to analyses took place.

c) In either case, changes in the analysis– including those made in response to peer review comments– should be identified as such in the Methods section of the paper, with rationale.

Thank you. We have added the following text (at the end of the Methods section since it refers to methods mentioned earlier in the text):

> **Study planning** Investigating asthma inequalities in Wales was a planned part of the first author's doctoral thesis about creating and utilising the Wales Asthma Observatory. Early results of the health service utilisation count regression models against the WIMD 2011 score quintile for an earlier follow-up period were previously published [9, 23]. To provide a more comprehensive picture about inequalities, the analysis was developed to include wider outcomes such as asthma emergency admissions, prescribing, and deaths as well as the WIMD 2011's individual domains as predictors. The GAMs were developed in response to peer review comments to investigate non-linear associations.

32. *The academic editor (AE) has some comments on one of the concerns of reviewer 3, please do take into account the view of the academic editor, this point should certainly be responded to by the authors in their rebuttal letter although as noted by the AE, the exact analysis proposed by the reviewer does not have to be added in revision.

Thank you. We have responded to the Reviewer 3's comment and added the limitation of measuring socioeconomic deprivation at the area level to the Discussion.

We are grateful for the opportunity to respond to this comprehensive, constructive feedback, which has helped to improve the quality of our work. We trust that these revisions are to your satisfaction. Please do not however hesitate to contact me if you

require any further clarification or revisions.

Sincerely,

Mohammad Al Sallakh

References

1 Schatz M, Zeiger RS, Vollmer WM, et al. The Controller-to-Total Asthma Medication Ratio Is Associated With Patient-Centered As Well As Utilization Outcomes. *Chest* 2006; **130**: 43–50.

2 Office for National Statistics. Analysis of death registrations not involving coronavirus (COVID-19), England and Wales: 28 December 2019 to 1 May 2020. URL: https://www.ons.gov.uk/peoplepopulationandcommunity/birthsdeathsandmarriages/deaths/articles/analysisofdeathregistrationsnotinvolvingcoronaviruscovid19englandandwales28december2019to1may2020/technicalannex (visited on June 5, 2020).

3 Gupta RP, Mukherjee M, Sheikh A, and Strachan DP. Persistent variations in national asthma mortality, hospital admissions and prevalence by socioeconomic status and region in England. *Thorax* 2018; **73**: 706–712.

4 Weinberg L, Ianno D, Churilov L, et al. Restrictive intraoperative fluid optimisation algorithm improves outcomes in patients undergoing pancreaticoduodenectomy: A prospective multicentre randomized controlled trial. *PLOS ONE* 2017; **12**: e0183313.

5 Jacobs R, Gutacker N, Mason A, et al. Determinants of hospital length of stay for people with serious mental illness in England and implications for payment systems: a regression analysis. *BMC Health Services Research* 2015; **15**.

6 Welsh Assembly Government. The Welsh Index of Multiple Deprivation (WIMD) 2011: Technical Report. Cardiff, 2011.

7 Newton S, Braithwaite D, and Akinyemiju TF. Socio-economic status over the life course and obesity: Systematic review and meta-analysis. *PLOS ONE* 2017; **12**: e0177151.

8 Lorant V. Socioeconomic Inequalities in Depression: A Meta-Analysis. *American Journal of Epidemiology* 2003; **157**: 98–112.

9 Al Sallakh MA. Creating and utilising the Wales Asthma Observatory to support health policy, health service planning and clinical research. PhD thesis. Swansea University, 2018.

10 Schatz M, Nakahiro R, Crawford W, Mendoza G, Mosen D, and Stibolt TB. Asthma quality-of-care markers using administrative data. *Chest* 2005; **128**: 1968–1973.

11 Schatz M and Zeiger RS. Improving asthma outcomes in large populations. *J Allergy Clin Immunol* 2011; **128**: 273–277.

12 Federman AD, Wolf MS, Sofianou A, et al. Self-Management Behaviors in Older Adults with Asthma: Associations with Health Literacy. *Journal of the American Geriatrics Society* 2014; **62**: 872–879.

13 Wang Y, Hunt K, Nazareth I, Freemantle N, and Petersen I. Do men consult less than women? An analysis of routinely collected UK general practice data. *BMJ Open* 2013; **3**: e003320.

14 Prescott E, Lange P, and Vestbo J. Effect of gender on hospital admissions for asthma and prevalence of self-reported asthma: a prospective study based on a sample of the general population. Copenhagen City Heart Study Group. *Thorax* 1997; **52**: 287–289.

15 Singh AK. Sex Differences Among Adults Presenting to the Emergency Department With Acute Asthma. *Archives of Internal Medicine* 1999; **159**: 1237.

16 Schatz M and Camargo CA. The relationship of sex to asthma prevalence, health care utilization, and medications in a large managed care organization. *Annals of Allergy, Asthma & Immunology* 2003; **91**: 553–558.

17 Miller MK, Lee JH, Blanc PD, et al. TENOR risk score predicts healthcare in adults with severe or difficult-to-treat asthma. *Eur Respir J* 2006; **28**: 1145–1155.

18 Schatz M, Clark S, and Camargo CA. Sex Differences in the Presentation and Course of Asthma Hospitalizations. *Chest* 2006; **129**: 50–55.

19 Zein JG and Erzurum SC. Asthma is Different in Women. *Current Allergy and Asthma Reports* 2015; **15**.

20 Leynaert B, Sunyer J, Garcia-Esteban R, et al. Gender differences in prevalence, diagnosis and incidence of allergic and non-allergic asthma: a population-based cohort. *Thorax* 2012; **67**: 625–631.

21 Sullivan K and Thakur N. Structural and Social Determinants of Health in Asthma in Developed Economies: a Scoping Review of Literature Published Between 2014 and 2019. *Current Allergy and Asthma Reports* 2020; **20**.

22 Institute of Health Equity. Health equity in England: the Marmot review 10 years on. London, 2020. URL: http://www.instituteofhealthequity.org/resources-reports/marmot-review-10-years-on.

23 Al Sallakh MA, Rodgers SE, Lyons RA, Sheikh A, and Davies GA. Socioeconomic deprivation and inequalities in asthma care in Wales. *The Lancet* 2017; **390**: S19.
